# OP25 Teleradiology Public-Private Partnership Models: Informing Health Technology Assessment Findings In India

**DOI:** 10.1017/S0266462325100834

**Published:** 2025-12-29

**Authors:** Anupama Sharma, Ranjan Choudhury, Anjaney Shahi

## Abstract

**Introduction:**

In India, the Free Diagnostic Service Initiative leverages teleradiology and technology to address the shortage of skilled radiologists, improving healthcare access. X-ray films are digitized for remote reporting, enhancing equitable healthcare delivery. The study explored teleradiology public-private partnership (PPP) models, emphasizing their synergy with artificial intelligence (AI) as a scalable solution to strengthen public health services in low- and middle-income countries.

**Methods:**

The study prospectively analyzed telereporting services under PPP models across Indian states and union territories and reviewed literature from January to July 2023. Data from Program Implementation Plans and Records of Proceedings were examined. The study assessed resources, expertise, and accountability using the World Health Organization’s Public-Private Mix Framework, which focused on Enabling Environment, Service Delivery, and Monitoring & Evaluation. It highlighted infrastructure challenges, skilled human resource needs, and impacts on access, equity, regulation, incentives, innovation, and uptake in radiology systems, emphasizing resource-intensive digitization (Digital Imaging and Communications in Medicine; Picture Archiving and Communication System). The findings provide insights into the distribution of responsibilities between public and private entities for effective healthcare delivery.

**Results:**

Teleradiology PPP services in India, implemented in 14 out of 36 states, were categorized into three models: outsourcing X-ray and telereporting, outsourcing digitization and telereporting, and outsourcing human resources and teleradiology services. States like Tamil Nadu and Gujarat used teleradiology for image enhancement and diagnosis. Teleradiology PPPs bridged urban-rural disparities, improving healthcare access for marginalized populations. These models reduced diagnostic costs, enhanced diagnostic accuracy, and improved patient satisfaction and treatment outcomes. They demonstrated scalability, resource optimization, and long-term feasibility. Policymakers are exploring further integration of AI into teleradiology to enhance radiodiagnosis and healthcare delivery across India.

**Conclusions:**

By incorporating evidence from PPP models into the Health Technology Assessment office of the Department of Health Research and the Indian Council of Medical Research under the Ministry of Health, states in India can now make informed decisions regarding the scaling, funding, and optimization of teleradiology services to meet the healthcare needs effectively.

